# PTTG regulates the metabolic switch of ovarian cancer cells via the c-myc pathway

**DOI:** 10.18632/oncotarget.5726

**Published:** 2015-10-26

**Authors:** Xiu Wang, Wanxing Duan, Xuqi Li, Jiangbo Liu, Donghong Li, Lianhong Ye, Lu Qian, Aijun Yang, Qinhong Xu, Han Liu, Qiaoshan Fu, Erxi Wu, Qingyong Ma, Xin Shen

**Affiliations:** ^1^ Department of Hepatobiliary Surgery, First Affiliated Hospital of Medical College, Xi'an Jiaotong University, Xi'an, 710061, China; ^2^ Department of Gynecology and Obstetrics, Affiliated Guangren Hospital of Xi'an Jiaotong University, Xi'an, 710004, China; ^3^ Department of Neurosurgery, Baylor Scott & White Health, Temple, Texas, 76502, USA; ^4^ Department of Anesthesiology, First Affiliated Hospital of Medical College, Xi'an Jiaotong University, Xi'an, 710061, China

**Keywords:** human pituitary tumor-transforming gene (PTTG), metabolic switch, ovarian cancer, aerobic glycolysis, oxidative phosphorylation

## Abstract

Human pituitary tumor-transforming gene (PTTG) is a proto-oncogene involved in the development, invasion, and metastasis of many types of cancer, including ovarian cancer. However, little is known about the role of PTTG in the metabolic shift of ovarian cancer cells. In our study, we show that PTTG expression was positively correlated with the differentiation degree of ovarian cancer tissue. In addition, PTTG suppression by specific shRNA could inhibit the proliferation of ovarian cancer cells A2780 and SKOV-3. Furthermore, aerobic glycolysis was suppressed and oxidative phosphorylation was increased in ovarian cancer cells after PTTG suppression. We further found that the expression of c-myc and several crucial enzymes involved in aerobic glycolysis (e.g., PKM2, LDHA, and glucose transporter 1 (GLUT-1)) were downregulated by PTTG knockwown. Overexpression of c-myc could prevent the metabolic shift induced by PTTG knockwown. Together, our findings suggest that the oncogene PTTG promotes the progression of ovarian cancer cells, and its loss resists tumor development, in part, by regulating cellular metabolic reprogramming that supports cell growth and proliferation via c-myc pathway.

## INTRODUCTION

Ovarian carcinoma is usually diagnosed at the advanced stage, and therefore it has a poor prognosis that ranks fourth among the gynecological malignancies and is the most deadly gynecological cancer, with a five-year survival rate of less than 30% [1–3]. Cancer cells share the common characteristics of rapid division and proliferation to generate essential biosynthetic building blocks, such as nucleic acids, amino acids, and lipids, all of which are a requirement of highly proliferative tumor cells [4]. Most of the cancer cells present an altered metabolic pattern compared with that of non-cancerous cells, which undergo aerobic glycolysis (Warburg effect). Tumor cells require much more glucose than non-cancerous cells even when there is abundant oxygen. This is in contrast to oxidative phosphorylation, which can produce more adenosine triphosphate (ATP) generated by glucose catabolism, aerobic glycolysis produces less ATP [5]. This metabolic switch can provide more intermediates for cell growth and division, and it is mainly regulated by both oncogenes and tumor suppressor gene mutants in a number of key cancer-promoting pathways [6, 7].

Human pituitary tumor-transforming gene (PTTG), known as securin, is a multifunctional proto-oncogene overexpressed in various tumors, including ovarian cancer [8–10]. Overexpression of PTTG *in vitro* induces cellular transformation and tumor development in transgenic mice [11]. The overexpression of PTTG is correlated with tumor invasion, progression, metastasis, and angiogenesis, suggesting that PTTG may play a crucial role in tumorigenesis [12–15]. Until now, little was known about the effects of PTTG on the metabolic switch and proliferation process of tumor cells.

In the present study, we show that the oncogene PTTG influences the aerobic glycolysis of ovarian cancer cells. Knockdown of PTTG can partly switch cancer cells from aerobic glycolysis to oxidative phosphorylation and reverse the metabolic phenotype of cancer cells.

## RESULTS

### The overexpression of PTTG is correlated with worse differentiation in ovarian cancer

**Figure 1:**
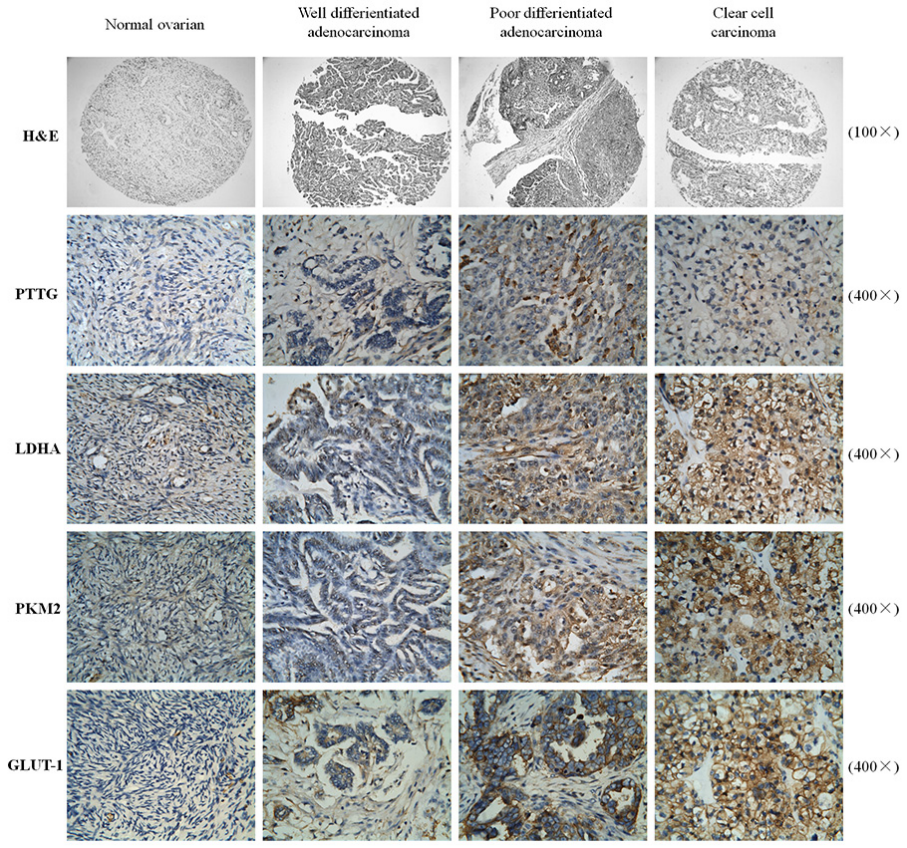
Analysis of the expression level of PTTG, as well as that of aerobic glycolysis-related enzymes PKM2, LDHA, and GLUT-1 in various differentiated ovarian carcinoma tissues For *H&E,* the magnification is 100×, for Immunohistochemical staining, the magnification is 400×.

We first compared the PTTG expression from different differentiated epithelial ovarian tissues via immunohistochemistry. The PTTG expression level in ovarian cancer tissue was correspondingly increased with worse tissue differentiation compared with normal ovarian tissue. The results show that there was a positive correlation between PTTG expression and the degree of epithelial ovarian cancer differentiation (Figure 1 and Supplementary Figure S1). These results indicate that oncogene PTTG may promote ovarian cancer growth and development.

It is known that cancer cells undergo aerobic glycolysis, which plays an important role during the process of cancer evolvement. Therefore, we determined the expression level of several enzymes involved in aerobic glycolysis, including LDHA, PKM2, and GLUT-1. The results show that an increase in PTTG levels is accompanied with an increase in LDHA, PKM2, and GLUT-1 expression, illustrating that PTTG may be involved in aerobic glycolysis in ovarian cancer. (Figure 1)

### PTTG knockdown inhibits ovarian cancer cells proliferation

**Figure 2:**
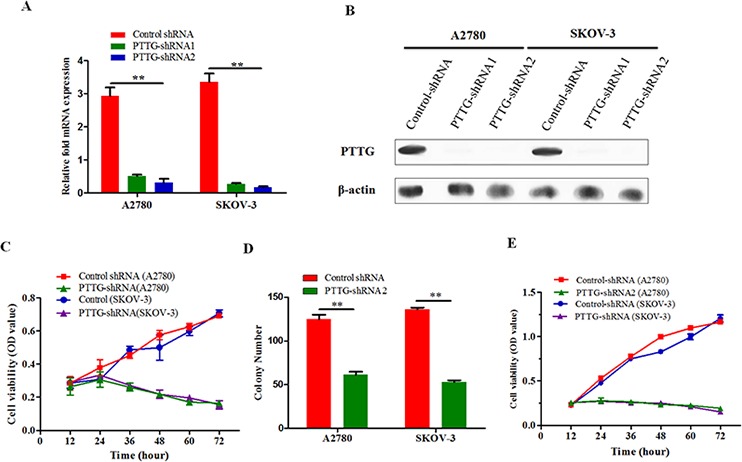
PTTG knockdown inhibits ovarian cancer cell proliferation **A.** After 24 h of PTTG-shRNA1 or PTTG-shRNA2 (lentivirus vector) transfection in ovarian cancer cells A2780 and SKOV-3, expression levels of PTTG were determined using real-time PCR and normalized to those of β-actin. **B.** 48 h post-transfection, PTTG expression levels were determined using Western blot. PTTG expression was also more evidently suppressed by PTTG-shRNA2 than by PTTG-shRNA1. **C.** Two stable ovarian cancer cells lines were transfected with lentivirus vector PTTG-shRNA2 and empty lentivirus vector and viability was assessed using MTT at 12 h, 24 h, 36 h, 48 h, 60 h, and 72 h. **D.** Colony formation was compared between PTTG-shRNA stable cells lines and empty vector cells (control shRNA), and PTTG-shRNA formed fewer colonies than control cells in the soft agarose culture. **E.** Analysis of PTTG-shRNA stably transfected cells and empty vector-transfected cell viability after the addition of 20 ng/ml EGF to the culture. The data represent the mean and SEM of triplicate experiments. **p < 0.01.

Next, we examined the roles of PTTG on the proliferation and colony formation of ovarian cancer. Lentivirus vector PTTG-shRNA1 and PTTG-shRNA2 were used to suppress PTTG expression in two ovarian cancer cell lines, A2780 and SKOV-3. From qRT-PCR and Western blotting results, we found that PTTG-shRNA2 is more effective than PTTG-shRNA1 in knocking down the PTTG gene (Figure 2A and Figure 2B). Accordingly, we chose to transfect A2780 and SKOV-3 with PTTG-shRNA2 (hereafter, PTTG-shRNA refers to PTTG-shRNA2). Flow cytometry approach was used to screen for stably transfected cells. At various time points after PTTG-shRNA transfection (12 h, 24 h, 36 h, 48 h, 60 h, and 72 h), the proliferation rate of A2780 and SKOV-3 were determined by MTT. The results show that PTTG knockdown inhibited the proliferation of both ovarian cancer cell lines (Figure 2C). The colony formation ability of both cell lines was evidently decreased, which was determined by soft agarose colony formation (Figure 2D). Epidermal growth factor (EGF), an upstream effector of PTTG, induces PTTG expression by a paracrine mechanism, leading to activation of the EGF receptor (EGFR) and promoted cancer cell proliferation [15, 16]. Therefore, we further tested whether PTTG knockdown in ovarian cancer cells still had proliferation ability after stimulation with EGF. The results show that EGF could not promote the growth of ovarian cancer cells in which PTTG expression had been silenced compared with control cells (Figure 2E).

### PTTG is required for aerobic glycolysis of ovarian cancer cells

Cancer cells need more nutrients for the synthesis of macromolecules, including nucleotides, amino acids, and lipids [17]. When cellular metabolism is altered by the aberrant expression of oncogenes or tumor suppressor genes, most cancer cells instead rely on aerobic glycolysis [4, 6, 7]. Aerobic glycolysis is an inefficient way to generate adenosine 5-triphosphate (ATP). However, this metabolic switch can provide more intermediates for biomass synthesis [18]. PTTG silence suppressed the proliferation of ovarian cancer cells and colony formation, but it is not known whether its effect on ovarian cancer is through its effect on aerobic glycolysis.

**Figure 3:**
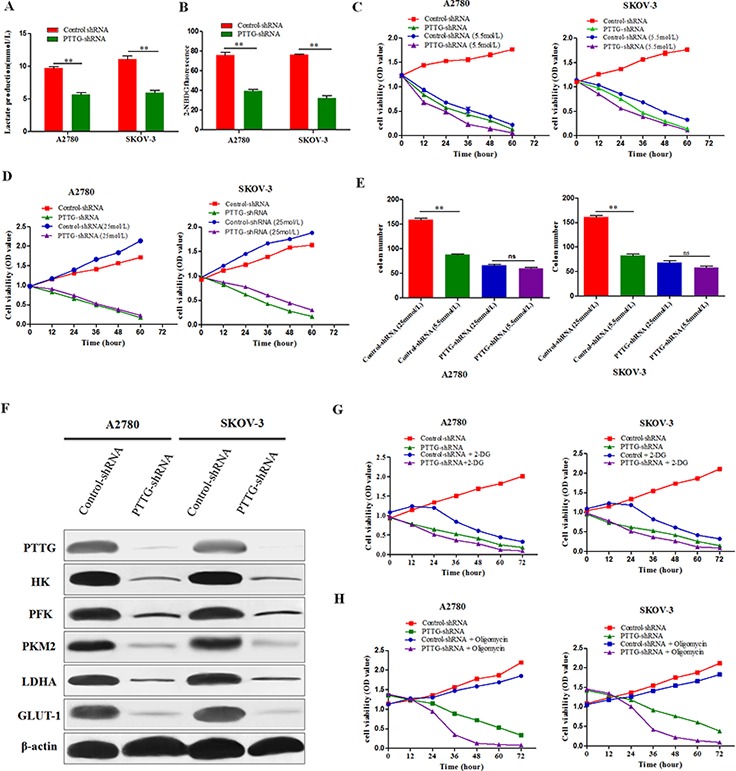
PTTG knockdown inhibits aerobic glycolysis in ovarian cancer cells **A.** Lactic acid production was compared between PTTG knockdown cells and control cells, and PTTG knockdown diminished lactic acid production. **B.** Glucose uptake was compared between PTTG knockdown cells and control cells, and PTTG knockdown cells showed decreased 2-NBDG uptake as determined via green fluorescence using flow cytometry. **C&D.** The proliferation ability of PTTG-shRNA-transfected A2780 and SKOV-3 cells in low sugar medium with 5.5 mmol/L or 25 mmol/L glucose was compared with control cells. The results showthat PTTG-shRNA ovarian cancer cells tolerate a lower glucose concentration than control cells. **E.** Comparison of PTTG-shRNA ovarian cancer cells with control cells in a soft agarose colony formation assay number with the 5.5 mmol/L and 25 mmol/L glucose in the cellular culture medium. **F.** Western blot analysis of the expression level of several enzymes involved in aerobic glycolysis, including HK (hexokinasse), PFK (phosphofructokinase), PKM2 (pyruvate kinase M2) and LDHA (lactate dehydrogenase), as well as glucose transport GLUT-1. **G.** Analysis of the viability of PTTG-shRNA cells and control cells via MTT after treatment with 2-DG (100 mM) to inhibit glycolysis. **H.** Analysis of the viability of PTTG-shRNA cells and control cells via MTT after using oligomycin (2 μg/ml) to inhibit mitochondrial OXPHOS. The data represent the mean and SEM of triplicate experiments. *p < 0.05, **p < 0.01.

To confirm the influence of PTTG on the aerobic glycolysis of ovarian cancer cells, we used PTTG-shRNA lentiviral stable -transfected cell lines, and then determined the lactic acid production and glucose uptake. The results show that PTTG knockdown decreased the production of lactic acid in A2780 and SKOV-3 (Figure 3A). We use a fluorescent glucose analog, (2-(N-(7-nitrobenz-2-oxa-1,3-diazol-4-yl) amino)-2-deoxyglucose) (2-NBDG) to determine glucose uptake. The results show that PTTG-deficient cells evidently have decreased glucose uptake compared with the control cells (Figure 3B). From the above results, we find that PTTG suppression can lead to a decreased requirement for glucose in ovarian cancer cells. We further assayed the effect of PTTG on the viability and colony formation ability of A2780 and SKOV-3 cells under low glucose culture condition (5.5 mmol/L) and high glucose condition (25 mmol/L). The results illustrate that glucose starvation has a less effect on A2780 and SKOV-3 cells with PTTG knockdown than control cells with empty vector (Figure 3C). There is little difference in the colony number of A2780 and SKOV-3 cells with PTTG knockdown grown on a soft agarose culture with a low or high glucose concentration (Figure 3D). In other words, ovarian cancer cell proliferation is more dependent on glucose concentration in culture, and PTTG suppression can make the ovarian cancer cells less dependent on glucose concentration in culture. These results demonstrate that PTTG silence can decrease the requirement for glucose in ovarian cancer cells and inhibit aerobic glycolysis of cancer cells. We further determined the expression level of several crucial enzymes involved in aerobic glycolysis process, including HK (hexokinasse), PFK (phosphofructokinase), PKM2 (pyruvate kinase M2), LDHA (lactate dehydrogenase A), and glucose transporter (GLUT-1) (Figure 3E). PTTG silence markedly reduced the expression level of HK, PFK, PKM2, LDHA, and GLUT-1. Therefore, we deduce that PTTG may inhibit aerobic glycolysis of ovarian cancer cells via downregulating crucial enzymes involved in aerobic glycolysis.

Furthermore, we used 2-deoxy-D-glucose (2-DG) to inhibit glycolysis and oligomycin to inhibit OXPHOS (oxidative phosphorylation) and then measured the proliferation of the cells by MTT. The results show that PTTG-silenced cells are more sensitive to the mitochondrial metabolism inhibitor oligomycin, but that the control cells are more sensitive to the glycolytic inhibitor 2-DG (Figure 3F). This phenomenon indicates that PTTG silence can lead to a metabolic shift of the ovarian cancer cells from aerobic glycolysis to mitochondrial OXPHOS.

### PTTG knockdown induces a metabolic shift of ovarian cancer cells to oxidative phosphorylation

**Figure 4:**
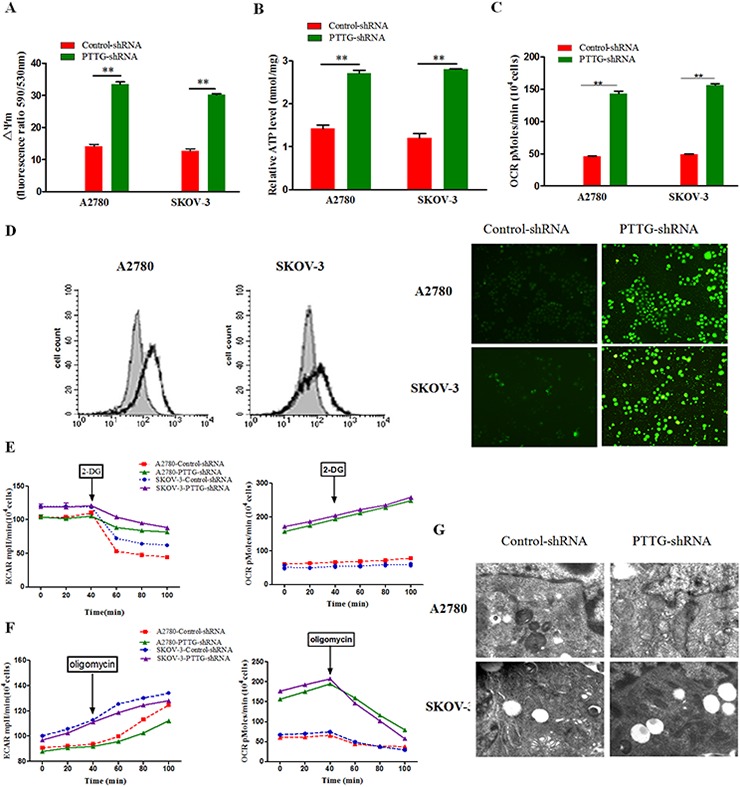
Oncogene PTTG loss promotes metabolic shift of ovarian cancer cells to oxidative phosphorylation **A.** Comparison of the alteration of the mitochondrial membrane potential (ΔΨm) of PTTG-shRNA cell line to control cells. The two ovarian cancer cell lines (A2780 and SKOV-3) exhibited increased mitochondrial membrane potential (ΔΨm) after PTTG knockdown. **B.** Analysis of the ATP production in the PTTG-shRNA cell line and control cells in the culture medium. The result show that when PTTG was silenced, the mitochondrial energy metabolism was improved in the two cell lines and ATP production increased. **C.** Oxygen consumption rate (OCR) as a key marker of mitochondrial OXPHOS. OCR was increased in the two ovarian cell lines (A2780 and SKOV-3). **D.** Analysis of reactive oxygen species (ROS) accumulation in the two ovarian cancer cell lines (A2780 and SKOV-3) after PTTG suppression, (left) fluorescence measurement determined using flow cytometry, (right) fluorescent assay under inverted reverse fluorescent microscope. **E & F.** Analysis of ECAR and OCR with time interval after 2-DG (100 mM) and oligomycin (2 μg/ml) treatment, respectively. **G.** Mitochondrial morphological alteration images under electron microscope. The data represent the mean and SEM of triplicate experiments. *p < 0.05, **p < 0.01.

From our study described above, PTTG knockdown can inhibit aerobic glycolysis of ovarian cancer cells. Therefore, we examined its effect on OXPHOS metabolism. First, we determined the ATP production and mitochondrial membrane potential of ovarian cancer cells. The results show that PTTG loss prominently increases ATP production and raises the mitochondrial membrane potential compared with empty vector-transfected ovarian cancer cells (Figure 4A, 4B). Glycolysis is inefficient for producing ATP, and most ATP is derived from OXPHOS [19, 20]. Therefore, we deduce that PTTG knockdown leads to a switch of the ovarian cancer cells from aerobic glycolysis to oxidative phosphorylation. Cancer cells exhibit more oxidative stress than normal cells. As a by-product of mitochondrial oxidative phosphorylation, mitochondria-derived reactive oxygen species (ROS) are markedly decreased. We measured the oxygen consumption rate (OCR) in A2780 and SKOV-3 cells stably transfected with PTTG-shRNA and observed that PTTG-silenced cells displayed enhanced oxygen consumption and decreased ROS production (Figure 4C, 4D). To assay glycolytic activity, we use 2-DG and oligomycin to suppress glycolysis and OXPHOS, respectively, and then the extracellular acidification rate (ECAR) was determined. The results show that the ECAR of control cells are more susceptible to the glycolysis inhibitor 2-DG than PTTG-silenced cells (Figure 4E). Furthermore, in PTTG-silenced cells, the OCR is more susceptible to the mitochondrial respiration inhibitor oligomycin than control cells (Figure 4F). In addition, we observed alteration in mitochondria morphology after PTTG silence under the electron microscope. The mitochondria of tumor cells usually have increased volume with a lucent swelling matrix, condensed configuration associated with disarrangement and distortion of cristae and partial or total cristolysis [21]. Here we observed that PTTG-silenced A2780 and SKOV-3 cells exhibited decreased mitochondrial volume, morphological homogeneity and clear cristae compared with control cells with empty vector (Figure 4G). These observations indicate that PTTG knockdown partly prompts metabolic shift from glycolysis to oxidative phosphorylation in ovarian cancer cells.

### Effect of PTTG on aerobic glycolysis and mitochondrial metabolism by activating the c-myc pathway

**Figure 5:**
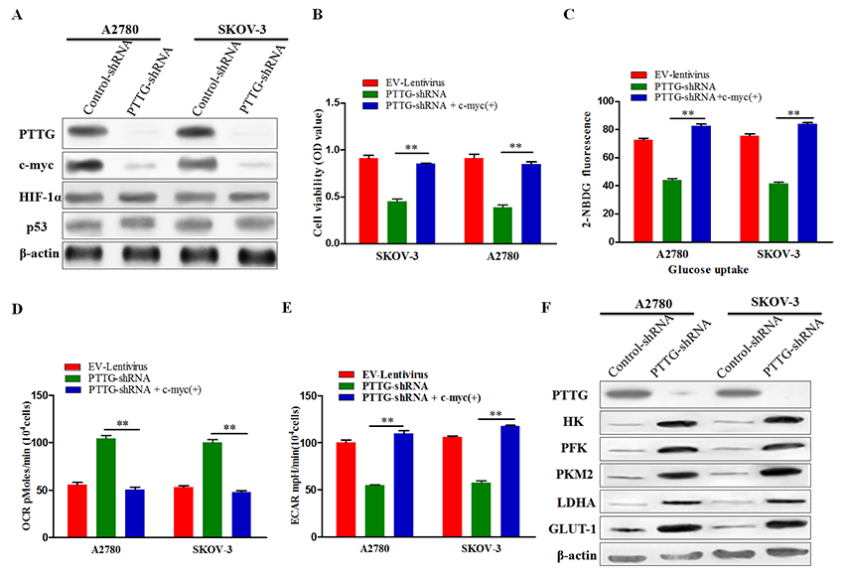
Effect of PTTG on aerobic glycolysis and mitochondrial metabolism by activating the c-myc pathway **A.** Analysis of c-myc, HIF-1, and p53 expression level via Western blot. **B.** Comparison of the alteration of cell proliferation of PTTG-shRNA-transfected A2780 and SKOV-3 cells before and after transfection with a c-myc-expressing lentivirus. **C.** Analysis of 2-NBDG fluorescence in PTTG-shRNA-transfected A2780 and SKOV-3 cells determined using flow cytometry before and after transfection of a c-myc-expressing lentivirus. **D & E.** OCR and ECAR were measured in PTTG-shRNA-transfected A2780 and SKOV-3 cells before and after transfection with a c-myc-expressing lentivirus. **F.** Western blotting analysis analysis the protein expression of several enzymes (HK, PFK, PKM2, LDHA, and GLUT-1) in PTTG-shRNA-transfected A2780 and SKOV-3 cells before and after transfection with a c-myc-expressing lentivirus vector.

Proto-oncogene c-myc, hypoxia-inducing factor 1 (HIF-1), and anti-oncogene p53 play indispensable roles in the metabolic mediating cascade. We surmised that the influence of PTTG on ovarian cancer cell metabolism phenotype transformation is mediated through its effects on c-myc, HIF-1, and p53. Therefore, we measured the expression level of c-myc, HIF-1, and p53 in PTTG-shRNA and empty vector stably transfected cell lines using Western blotting. The results show that the c-myc expression in A2780 and SKOV-3 was lowered after PTTG knockdown, while little change was observed with HIF-1 and p53 (Figure 5A). From these results, we initially speculated that PTTG regulated the metabolism of cancer cells perhaps through the c-myc pathway. To test our hypothesis, we used a lentivirus vector to induce overexpression of c-myc in PTTG-silenced cell lines. Then the cell proliferation, glucose uptake, OCR, and ECAR were determined. The results show that PTTG-silenced cells had enhanced proliferation (Figure 5B), glucose uptake (Figure 5C), decreased OCR (Figure 5D), and increased ECAR (Figure 5E) after transfected with the c-myc gene. We then determined the expression level of several enzymes (HK, PFK, PKM2, and LDHA) and GLUT-1, all of which are involved in glycolysis. Similarly, the expression levels of HK, PFK, PKM2, LDHA, and GLUT-1 were prominently increased (Figure 5F). These results illustrate that c-myc overexpression can reverse the effect of PTTG silence on aerobic glycolysis and mitochondrial metabolism, further demonstrating that the effect of PTTG on the metabolic reprogramming of ovarian cancer cells is mediated by the c-myc pathway.

## DISCUSSION

PTTG was originally isolated from rat pituitary tumors by Pei and her colleagues [22], Thereafter, extensive researches on the function of this gene was performed in relation to its overexpression in several cancer types [23–27]. PTTG is highly expressed in ovarian cancer, suggesting that PTTG may function in ovarian tumorigenesis. Understanding the molecular and functional mechanisms of PTTG and its important role in tumorigenesis in various cancers, including ovarian cancer, is of great interest.

In this study, we find that PTTG expression is positively correlated with the degree of ovarian cancer tissue differentiation. In other words, less differentiated ovarian cancer presents with a higher PTTG expression level. Here, we demonstrate that silence of the PTTG oncogene inhibited the proliferation of ovarian cancer cells and colony formation and interrupted the upstream EGF pathway that promotes ovarian cancer proliferation.

Oncogenes play an important role during the process of metabolism reprograming of cancer cells. Highly proliferating cancer cells, unlike most normal cells, which completely catabolize glucose by oxidative phosphorylation, require an altered glycolysis pathway. Even in an environment with copious oxygen, this alteration of metabolism shifts from oxidative phosphorylation to aerobic glycolysis, promoting the efficient conversion of glucose into the macromolecules needed to construct a new cell. Meanwhile, the shift to aerobic glycolysis is coupled with increased glucose uptake, lactate production, and ATP production. Our study shows that PTTG knockdown decreased lactic production and reduced glucose uptake of ovarian cancer cells. In contrast, the ATP production is increased. Furthermore, we found that the expression levels of several key enzymes involved in aerobic glycolysis, HK, PFK, PKM2, and LDHA, and glucose transport membrane protein GLUT-1 are evidently decreased. These results demonstrated that PTTG silence can inhibit the aerobic glycolysis of ovarian cancer cells, perhaps through downregulating several enzymes that involved in the glycolytic process. According to the Warburg effect, cancer cells with a reprogrammed metabolic pathway have a prior reliance on a high level of aerobic glycolysis as the major source of biomass macromolecular synthesis. As for the role of mitochondrial oxidative phosphorylation, until now, it was not fully understood. To further confirm the influence of PTTG in the OXPHOS process, we determined the role of mitochondria in OXPHOS function. The results show that PTTG knockdown can increase the mitochondrial potential (ΔΨm) and cellular oxygen consumption of ovarian cancer cells and decrease ROS and mitochondrial volume. The extracellular acidification rate (ECAR) of ovarian cancer cells was lowered; in other words, the glycolysis rate was suppressed. These results illustrate that PTTG knockdown reliably boosts mitochondrial OXPHOS while simultaneously inhibiting aerobic glycolysis metabolism.

To explore PTTG mediated intrinsic pathway, we silenced PTTG expression, and the expression level of several important upstream genes of aerobic glycolysis, including c-myc, HIF-1, and p53 were determined. The results show that after silencing of PTTG, the expression level of c-myc was significantly lowered, but HIF-1α and p53 expression levels showed little change. To further confirm the relationship or interaction between PTTG and c-myc during the process of aerobic glycolysis, we utilized c-myc overexpressing lentivirus to express the c-myc gene in PTTG-silenced ovarian cancer cells. The results demonstrated that c-myc overexpression reverses the proliferation, glycolysis and OXPHOS metabolism in the PTTG-silenced cells, and the expression level of several key enzymes in glycolysis is increased. All of these results illustrated that c-myc is a crucial factor during the PTTG pathway.

## MATERIALS AND METHODS

### Tissues source and immunohistochemistry

The ovarian tissue microarray consisting 8 normal samples, 102 ovarian cancer samples (the detail information was shown in Supplementary Table S1) were purchased from Alenabio (Xi'an, China). In addition, the histological differentiation and histological type of ovarian cancer samples were assessed by experienced pancreatic pathologists. Immunohistochemical staining for PTTG, LDHA, PKM2 and GLUT-1 were carried out using standard procedures as previously described [28]. PTTG staining was scored in accordance by two investigators with previous protocols as negative (0), weak (1+), moderate (2+), or strong (3+). Scoring was performed blindly with respect to the histologic grade of ovarian cancer specimens.

### Cell culture

The human ovarian carcinoma cell lines A2780 and SKOV-3 were obtained from ATCC (Manassas, VA, USA) and provided by Animal Lab Center of The Fourth Military Medical University, respectively. The two cell lines were cultured in high glucose DMEM supplemented with 10% FBS, 100 units/ml penicillin, and 100 mg/ml streptomycin. The cells were incubated at 37°C in 5% CO_2_. The growth medium was replaced every 3 days, and the cells were sub-cultured when they reached 80–90% confluence.

### Cell proliferation assay

Cell proliferation was measured using the MTT (3-(4,5-dimethy lthiazol-2-yl)-2,5-diphenyl tetrazoliumbromide) assay (Biotime). The cells were seeded into 96-well plates at a density of 5 × 10^3^ cells per well and incubated overnight in 10% FBS medium. After incubation for 12 h, 24 h, 36 h, 48 h, 60 h, and 72 h at 37°C, 5%CO_2_, the cell proliferation rate was determined using the MTT assay. Then, 20 μl MTT solution (5 mg/ml in distilled water) was added to each well, and the cells were incubated for 4 h at 37°C, after which the medium was removed. Then, 150 μl DMSO was added, and the samples were vibrated for 10 min. The absorbance at 490 nm was measured using a microplate reader. The results were the mean values and standard deviation (SD) from the experiments conducted in triplicate.

### qRT-PCR

Total cellular RNA was extracted using TRIzol Reagent (Invitrogen), qualified via ultraviolet spectrophotometer, then preserved in −80°C for future assays. Target gene expression was determined via the Bio-Rad Real-time PCR instrument using quantitative real-time PCR analysis (SYBR Two-step qRT-PCR kit, Takara), with equal amounts of cDNA being loaded into three duplicate wells. Relative copy numbers were calculated from a five-point standard curve generated from a serial dilution of cDNA. The expression of each gene was normalized to β-actin. The conditions for all qRT-PCR reactions were as follows: 10 min at 95°C followed by 30 s at 95°C, 1 min at 65°C, and 30 s at 72°C for 30 cycles. All PCR products were confirmed by the presence of a single peak upon melting curve analysis.

### Western blotting analysis

The total cellular proteins were extracted using RIPA lysate and qualified using a BCA kit (Biotime). Then, protein samples were separated using 12% SDS-PAGE and transferred to a nitrocellulose membrane. Blots were then probed with monoclonal anti-β-actin (working concentration 1:800, Sigma), monoclonal anti-PTTG (working concentration 1:200, Santa Cruz), monoclonal anti-HK (working content 1:200, Santa Cruz), monoclonal anti-PFK (working concentration 1:400, Santa Cruz), monoclonal anti-LDHA (working concentration 1:500, Santa Cruz), anti-pkm2 (working concentration 1:400, Santa Cruz), and monoclonal anti-GLUT-1 (working concentration 1:300, Santa Cruz) antibodies overnight at 4°C. Membranes were washed with TBS containing 0.05% Tween-20 (TBS-T) and then incubated with peroxidase-conjugated anti-mouse and anti-rabbit secondary antibodies. Protein bands were visualized using an enhanced chemiluminescence (ECL) system according to the manufacturer's instructions.

### shRNA and generation of stable cell lines

The pRNAT-U6.2/Lenti vector was purchased from Shanghai Gene Pharma Co. Ltd (Shanghai, China) and was used to express a shRNA targeting the PTTG gene. PTTG-shRNA1 targets the sequence GAGATCTCAAGTTTCAACA, PTTG-shRNA2 targets the sequence CTCTCATGATCCTTGACGA, and control shRNA targets the sequence GTTCTCCGAACGTGTCACGT (the sequences were shown in Supplementary Figure S2). A lentiviral packaging kit was used to harvest shRNA lentivirus (concentration 4 × 10^9^ TU/ml). All procedures were carried out according to the manufacturer's instructions. Then, A2780 (MOI 50) and SKOV-3 (MOI 35) were transfected with shRNA lentivirus. After 48 hours post-transfection, stable cell lines were generated via flow cytometric green fluorescence selection. Furthermore, PTTG knockdown was confirmed using qRT-PCR and Western blot assay.

### Soft agar colony formation assay

For the soft agar colony formation assay, 1.2% low melting-point agarose and 2 × DMEM were mixed at a 1:1 volume ratio. Then, 1.4 ml per well of 0.6% basal agar was added to 6-well plates and was allowed to solidify at room temperature. The cells were trypsinized into a single-cell suspension at a concentration of 1 × 10^4^/ml. Then, 0.6% low melting-point agarose and 2 × DMEM were mixed at a 1:1 volume ratio, and 1 ml of 0.3% upper agar and 100 μl of the single-cell suspension were added and allowed to solidify at room temperature. The plates were incubated at 37°C, 5% CO_2_ for 14 days. Colonies with a diameter bigger than 150 μm were counted under an inverted microscope.

### Measurement of lactate production

A2780 and SKOV-3 PTTG knockdown cells and control cells were seeded into 96-well plates at 5.0 × 10^3^ cells per well and incubated 37°C in a 5% CO_2_ for 12 hours. Then, the lactate concentration in the culture medium was measured with a microplate reader using a Lactate Assay kit (Abnova) according to the assay protocol. Fluorescence was assayed at the excitation/emission of 535 nm/590 nm.

### Glucose uptake assay

Cellular glucose uptake was measured using 2-[N-(7-nitrobenz-2-oxa-1,3-diazol-4-yl)amino]-2-deoxy-D-glucose(2-NBDG; Invitrogen) and flow cytometric detection of the fluorescence produced by the cells. A2780 and SKOV-3 PTTG knockdown cells and control cells were seeded into 6-well plates at 1 × 10^6^ cells per well. After the cells achieved adherence, the cells were washed twice with PBS, and serum-free medium and 2-NBDG was added at a final concentration of 10 μM; the cells were then incubated for 30 min at 37°C. The 2-NBDG uptake reaction was stopped by removing the incubation medium and washing the cells twice with PBS. Green fluorescence was observed under a fluorescence microscope, cells were harvested, and 5 μl per well of 7-amino-actinomycin (7-AAD) dye was added; fluorescence was further quantified via FACScan flow cytometry (BD Bioscience).

### Glucose starvation

Glucose starvation was performed by washing adherent cells with PBS, incubating the cells in low-glucose DMEM, and then assaying cellular proliferation at 12 h, 24 h, 36 h, 48 h, 60 h, and 72 h using MTT. Soft agar colony formation was assayed in the low-glucose DMEM (5 mM glucose) agarose culture medium supplemented with 20% FBS. Low-glucose DMEM was used for soft agarose preparation, and the other procedures have been previously described.

### Mitochondrial membrane potential measurement

Mitochondrial transmembrane potential (Δψm) was detected using a JC-1 mitochondrial membrane potential assay kit (Beyotime) following the manufacturer's protocol. The four ovarian carcinoma stable cell lines (including A2780 and SKOV-3 with PTTG knockdown and two empty vector cell lines) were incubated in 96 cell culture plates at 1.5 × 10^4^ cells/well. The cells were then resuspended in 100 μl staining liquid (10 μg/ml of JC-1), a lipophilic cationic probe, incubated at 37°C for 20 min, washed twice with staining buffer, and then placed in fresh serum-free medium. Fluorescence was measured using a microplate reader at the excitation/emission of 488 nm/530 nm. All experiments were performed in triplicate, and all values were expressed as the mean ±standard error of the mean (SEM).

### ATP qualification

ATP production of cell lysates was assayed by fluorescence microplate reader at the excitation/emission of 480 nm/520 nm using an ATP assay kit (Beyotime) following the manufacturer's protocol.

### Measurements of OCR and ECAR

The oxygen consumption rate (OCR) and the extracellular acidification rate (ECAR) were measured in real time using a Seahorse Bioscience XF24 extracellular flux analyzer. A2780 and SKOV-3 with PTTG knockdown and two empty vector cell lines were seeded at 6 × 10^4^ cells/well in a 24-well plate and equilibrated with DMEM lacking bicarbonate at 37°C for 1 hour in an incubator lacking CO_2_. OCR and ECAR readings were taken using a 2 minute mix, 1 minute wait, and 2 minute read cycling protocol. All the XF24 data were reported as OCR or ECAR values normalized to cell counts. Measurements are reported in pmol/min for oxygen consumption and mpH/min for extracellular acidification rate. Each experiment was performed at least three times.

### Glycolysis AND mitochondria respiration inhibition assays

The cellular proliferation assay was performed in the presence of a glycolysis inhibitor or a mitochondria respiration inhibitor. Cells were seeded at 1.0 × 10^4^ cells/well in 96-well plates, were treated with glycolysis inhibitor 2-DG (100 mM) or an inhibitor of mitochondrial respiration oligomycin (2 μg/ml), and incubated 4 h at 37°C in 5% CO_2_. The cell proliferation rate was determined using MTT with Microplate Reader.

### Electron microscopy assays

Cells were trypsined from 6-well plates; 1.5 × 10^5^ cells were fixed in 2.5% glutaraldehyde in 0.1 M sodium cacodylate buffer for 2 h at 4°C, and then postfixed in 1% osmium tetroxide. The cells were then dehydrated in a graded series of ethanol and embedded in LX112. Thin sections (60 nm) were cut, stained with uranyl acetate and lead citrate, and mitochondrial morphologic alterations were observed under an electron microscope.

### Reactive oxygen species (ROS)

Intracellular reactive oxygen species (ROS) production was measured using the fluorescence probe, DCFH-DA (Reactive Oxygen Species Assay Kit, Biotime) according to the manufacturer's protocol. DCFH-DA penetrates the cellular membrane, and it is then hydrolyzed into DCFH without fluorescence by esterase. DCFH can be oxidized by intracellular ROS-producing DCF with fluorescence. Then, 2 × 10^6^ A2780 and SKOV-3 with PTTG knockdown and control cells were harvested, and serum-free culture medium with DCFH-DA diluted at a final concentration of 10 μM was added for 20 min in the dark at 37°C and reversed at 5 min intervals. Before being assayed, the cells were washed twice with PBS, and ROS was measured using FACSCalibur (BD Biosciences) with argon laser excitation at 488 nm.

## CONCLUSION

In conclusion, proto-oncogene PTTG plays an important role during the initiation of the ovarian cancer metabolism switch via the c-myc pathway, effecting downstream targets of c-myc, which include several key enzymes involved in aerobic glycolysis. PTTG further alters the cancer cells' metabolic phenotype and influences tumor cell proliferation and tumor growth. Thus, we provide the evidence that PTTG should be considered to be a potential target for anticancer therapy.

## SUPPLEMENTARY FIGURES AND TABLE


