# Supplementary material for: PTTG regulates the metabolic switch of ovarian cancer cells via the c-myc pathway
Source: Oncotarget. 2015 Oct 26;6(38):40959–69. doi: 10.18632/oncotarget.5726 (PMC4747382; doi:10.18632/oncotarget.5726)
Supplement: Supplementary file 1 [file oncotarget-06-40959-s001.pdf]

SUPPLEMENTARY FIGURES AND TABLE

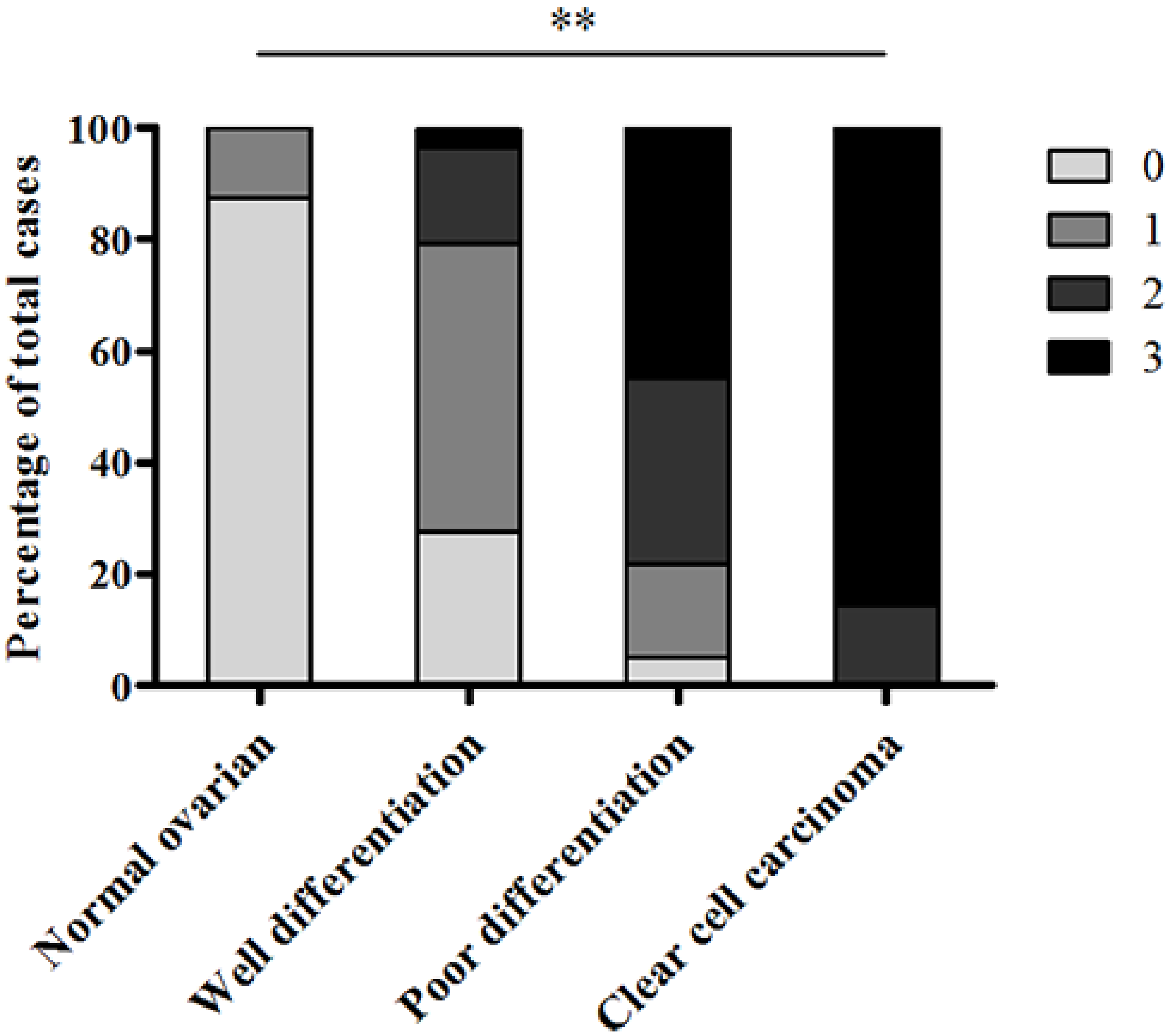

Supplementary Figure S1: The statistical result of PTTG expression in normal ovarian and different differentiated ovarian cancer tissues by immunohistochemistry, ****P* < 0.01.**

shRNA sequences for PTTG

PTTG-shRNA1:

Targeted sequence:

**GAGATCTCAAGTTTCAACA**	**TGTTGAAACTTGAGATCTC**

HpaI sense seqTence (19nt) Loop antisense seqTence (19nt) XhoI

5’-TG GAGATCTCAAGTTTCAACA **TTCAAGAGA**TGTTGAAACTTGAGATCTC TTTTTT C-3’

3’-AC CTCTAGAGTTCAAAGTTGT **AAGTTCTCT** ACAACTTTGAACTCTAGAG AAAAAA GAGCT-5’

PTTG-shRNA2:

Targeted sequence:

**CTCTCATGATCCTTGACGA**	**TCGTCAAGGATCATGAGAG**

HpaI sense seqTence (19nt) Loop antisense seqTence (19nt) XhoI

5’-TG CTCTCATGATCCTTGACGA**TTCAAGAGA**TCGTCAAGGATCATGAGAGTTTTTT C-3’

3’-AC GAGAGTACTAGGAACTGCT**AAGTTCTCT**AGCAGTTCCTAGTACTCTCAAAAAA GAGCT-5’

The structure of pRNAT-U6.2/Lenti vector

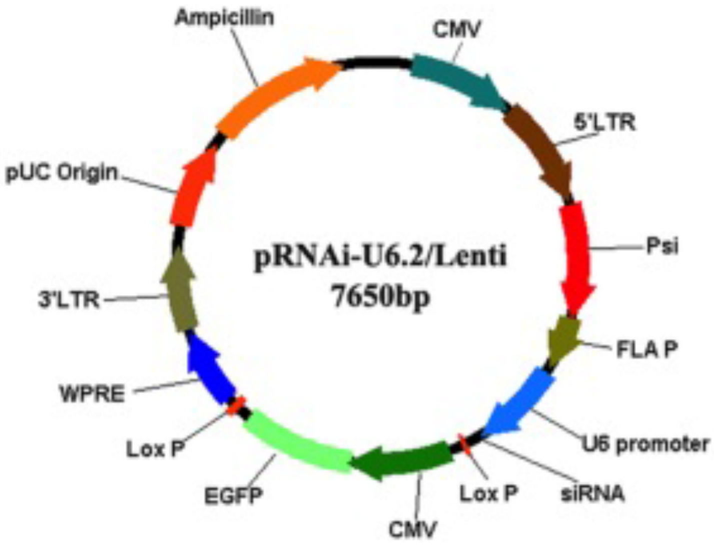

Supplementary Figure S2: The shRNA sequences for PTTG and the structure of pRNAT-U6.2/Lenti vector.

Supplementary Table S1: The clinicopathologic informations of patients with ovarian carcinoma

Parameters	Case (percentage)
**Age** (mean51; range: 25–79)	
≤60	87 (85.3%)
>60	15 (14.7%)
**Clinical stage** (FIGO staging system)	
I	53 (52.0%)
II	19 (18.6%)
III	24 (23.5%)
IV	6 (5.9%)
**Grade**	
Low	29 (28.4%)
High	67 (65.7%)
N/A	6 (5.9%)
**Histology**	
Serous	75 (73.5%)
Mucinous	12 (11.8%)
Clear cell	8 (7.8%)
Other	7 (6.9%)

N/A, not available.
Other, including mixed epithelial tumors and carcinomas not otherwise specified.
